# *Lactobacillus salivarius* SNK-6 Regulates Liver Lipid Metabolism Partly via the miR-130a-5p/MBOAT2 Pathway in a NAFLD Model of Laying Hens

**DOI:** 10.3390/cells11244133

**Published:** 2022-12-19

**Authors:** Lihui Zhu, Rongrong Liao, Jiwen Huang, Changfeng Xiao, Yunzhou Yang, Huiying Wang, Daqian He, Huaxiang Yan, Changsuo Yang

**Affiliations:** 1 Institute of Animal Husbandry and Veterinary Science, Shanghai Academy of Agricultural Sciences, Shanghai 201106, China; 2 National Poultry Research Center for Engineering and Technology, Shanghai 201106, China; 3 College of Animal Science and Technology, Zhejiang Agriculture and Forestry University, Hangzhou 311300, China

**Keywords:** *Lactobacillus salivarius* SNK-6, fatty liver, fat deposition, PPAR/SREBP pathway

## Abstract

*Lactobacillus* spp., as probiotics, have shown efficacy in alleviating nonalcoholic fatty liver disease (NAFLD). Here, we screened a new probiotic strain, *Lactobacillus salivarius* SNK-6 (*L. salivarius* SNK-6), which was isolated from the ileum of healthy Xinyang black-feather laying hens in China. We investigated the beneficial activity of *L. salivarius* SNK-6 in a NAFLD model in laying hens and found that *L. salivarius* SNK-6 inhibited liver fat deposition and decreased serum triglyceride levels and activity of aspartate transaminase and alanine transaminase. MBOAT2 (membrane-bound O-acyltransferase domain containing 2) was directly targeted by miR-130a-5p, which was downregulated in the liver of NAFLD laying hens but reversed after *L. salivarius* SNK-6 treatment. Downregulation of MBOAT2, *L. salivarius* SNK-6 supplementation in vivo, and *L. salivarius* SNK-6 cell culture treatment in vitro suppressed the mRNA expression of genes involved in the PPAR/SREBP pathway. In addition, 250 metabolites were identified in the supernatants of *L. salivarius* SNK-6 culture media, and most of them participated in metabolic pathways, including amino acid, carbohydrate, and lipid metabolism. Targeted metabolomic analysis revealed that acetate, butyrate, and propionate were the most abundant short-chain fatty acids, while cholic acid, ursodeoxycholic acid, chenodeoxycholic acid, and tauroursodeoxycholic acid were the four most-enriched bile acids among *L. salivarius* SNK-6 metabolites. This may have contributed to the reparative effect of *L. salivarius* SNK-6 in the NAFLD chicken model. Our study suggested that *L. salivarius* SNK-6 alleviated liver damage partly via the miR-130a-5p/MBOAT2 signaling pathway.

## 1. Introduction

Nonalcoholic fatty liver disease (NAFLD) is a major form of liver disease worldwide. It is characterized by excessive lipid accumulation, which causes liver inflammation and mitochondrial dysfunction; all of which contribute to accelerated liver damage [1,2]. Although diet/exercise may confer some benefits for NAFLD, there is currently no specific drug for NAFLD. Recently, probiotics have attracted a lot of attention as a potential NAFLD treatment, especially with the discovery that gut microbes are involved in the pathogenic process of NAFLD [1]. Probiotics are a class of live microorganisms that are beneficial to the host, including improving the microecological balance and health of the host [3]. As functional foods, probiotics have been isolated from a variety of sources and studied for their benefits to health and a wide range of diseases. Evidence suggests that probiotic intake is beneficial for gut microbiota composition normalization in patients with NAFLD, which in turn may improve intestinal barrier function and reduce liver inflammation. Symbiotic restoration of the gut microbiota also benefits the production of microbiota-derived metabolites with known health benefits, such as short-chain fatty acids (SCFAs) [4]. *Lactobacillus salivarius* (*L. salivarius*) is effective in alleviating liver damage [5,6]. One of its mechanisms is to reshape the intestinal flora, enhance the intestinal barrier function, and regulate liver lipid metabolism [6,7,8]. However, little is known about the therapeutic effect of *L. salivarius* on NAFLD. Similar to NAFLD, fatty liver hemorrhagic syndrome (FLHS) is a common noninfectious disease that is characterized by excessive liver triglyceride (TG) deposition, accompanied by hemorrhage, causing huge economic loss in commercial poultry production [9]. Like humans, the liver is the major site for de novo lipid synthesis in chickens [10]. Increased de novo lipid synthesis, oxidation retardation of free fatty acids, and insulin inhibition were also observed in FLHS chicken models [11,12]. Recently, chicken fatty liver was considered to be a good model for human NAFLD research because the genetic makeup of chicken is approximately 70% homologous to that of humans and pathogenesis of FLHS is similar to that of NAFLD in humans [13,14,15]. For example, Qiu et al. used layers as a preclinical model to study the effect of pyrroloquinoline quinone on reducing metabolic dysfunction related fatty liver disease [14]. Plasma acetoacetyl-CoA synthetase, dipeptidyl-peptidase 4, glutamine synthetase, and glutathione S-transferase concentrations are well-established biomarkers for NAFLD, which were selected from the plasma of the NAFLD model of laying hens and reported to be related to liver fibrosis and liver injury [15]. All these results suggested that adult chickens with age-associated steatosis similar to human NAFLD are an attractive animal model.

A lactic acid bacterium, *Lactobacillus salivarius* SNK-6 (*L. salivarius* SNK-6), was isolated from the ileum of healthy Xinyang black-feather laying hens and used to investigate whether *L. salivarius* SNK-6 supplementation alleviated liver lipid accumulation in a NAFLD model in laying hens, and the potential regulatory mechanism. Our data may provide a new probiotic strain for liver disease treatment.

## 2. Materials and Methods

### 2.1. Genome Sequencing of Bacteria

*L. salivarius* SNK-6 was isolated from the ileum of Xinyang black-feather laying hens and grown in De Mann, Rogosa and Sharp broth, and incubated at 41 °C for 24 h under anaerobic conditions. Whole-genome sequencing of *L. salivarius* SNK-6 was performed on the PacBio (Pacific Biosciences, Menlo Park, CA, USA) and Illumina MiSeq (Illumina, San Diego, CA, USA) sequencing platforms. SOAPec v2.0 software was used to assemble paired-end reads de novo into high-quality sequences [16]. Clean reads were assembled using SPAdes genome assembler v3.11.1 [17]. PacBio reads were assembled using HGAP and CANU (v1.6) software to obtain scaffold sequences [18,19]. The contigs and scaffolds sequences obtained from the two platforms were assembled and the inner gaps within each scaffold were filled by pilson v1.22 software [20]. Putative coding sequences were annotated with GeneMarkS software. tRNA and rRNA were annotated by tRNAscan-SE and Barrnap (0.9-dev) (http://github.com/tseemann/barrnap (accessed on 3 March 2022)), respectively. The complete genome sequence of *L. salivarius* SNK-6 is available at GenBank under accession number CP011403-CP011405. The strain was deposited at the Chinese Center for Type Culture Collection, accession number CCTCC No: M2018044, Wuhan, China).

### 2.2. Animal Models

One hundred and eighty 40-week-old healthy Xinyang black-feather laying hens of similar weight (Shanghai Poultry Breeding Co. Ltd., Shanghai, China) were randomly divided into two groups with five replicates in each group, and each replicate had 18 chickens. The 2 groups were defined as the fatty liver group fed with the basic diet containing 16.5% crude protein and 2700 kcal/kg metabolizable energy, and the *L. salivarius* SNK-6 group fed the basal diet supplemented with 10^8^ CFU/kg *L. salivarius* SNK-6 in water. The trial period was 12 weeks, of which the first week was the pretrial period. At 52 weeks of age, three chickens in each replicate (15 chickens in each group) were selected randomly, and serum and liver samples were collected for further analysis. In addition, serum and liver samples from 12 laying hens aged 25 weeks with no liver hemorrhage were used as the negative control group.

### 2.3. Histological Analysis

The chicken liver tissue samples were fixed in 4% paraformaldehyde, embedded in paraffin, and then cut into 4-μm-thick pieces. Hematoxylin–eosin staining was performed and the sections were observed under an optical microscope (Nikon Corporation, Tokyo, Japan).

### 2.4. Oil-Red O Staining

Liver samples were embedded in Tissue-Tek OCT embedding compound, frozen on dry ice, cut into 8-μm-thick frozen sections, and stained with an Oil Red Staining kit (Solarbio, Beijing, China). LMH cells were cultured with 1 mM free FAs (FFAs), as we described previously [21], and a 10-fold dilution of *L. salivarius* SNK-6 medium supernatant was added and incubated for 24 h. Cells were collected and fixed in 4% paraformaldehyde, stained by Oil Red O Staining kit (Solarbio), and observed under an electron microscope (Nikon). Data were analyzed by Image-Pro Plus 6.0 software. The staining positive area and total area of sections were calculated.

### 2.5. Detection of Lipid Levels and Enzymatic Activity

The levels of total cholesterol (TCHO) and TG, and the activity of aspartate transaminase (AST) and alanine transaminase (ALT) in the serum were detected using commercial kits (NanJing JianCheng Bioengineering Institute, Nanjing, China).

### 2.6. Supernatants Collection

Supernatants were collected when the cells had a minimum cell number of 10^8^ CFU/mL. Then the cell-free supernatants isolated by centrifugation at 5000 g for 30 min and stored at −80 °C until analysis.

### 2.7. Cell Culture and Transfection

LMH cells from the American Type Culture Collection (Manassas, VA, USA) were cultured in DMEM/F12 containing 10% fetal bovine serum, streptomycin (100 mg/mL), and penicillin (100 mg/mL) at 37 °C in an atmosphere of 5% CO_2_. Cells (1.5 × 10^5^/mL) were seeded in 6-well plates and transfected with mimics, inhibitors, scrambled miRNA, siRNA-MBOAT2 (membrane-bound O-acyltransferase domain containing 2), and control siRNA (40 nM, 10 pmol/mL) using Lipofectamine™ 2000 (Life Technologies, Carlsbad, CA, USA). The oligonucleotides were synthesized by Sangon Biotech Co. Ltd. (Shanghai, China) (Table S1). Cells were collected after 24 h and 48 h post-transfection for RT-qPCR and Western blotting analysis, respectively.

### 2.8. Dual Luciferase Reporter Assay

The wild and mutated type sequences of target 3′ untranslated region (UTR) with miR-130a-5p binding sites were synthesized and cloned into the psiCHECK-2 (Promega, Madison, WI, USA). Plasmids were transfected into HEK293T cells using Lipofectamine™ 2000 (Life Technologies). The Dual Glo Luciferase assay system (Promega) was used to measure luciferase activity.

### 2.9. Quantitative Real-Time RT-PCR

Total RNA was extracted using TRIzol reagent (Life Technologies) and reverse transcribed using the miScript II RT Kit (QIAGEN GmbH, Hilden, Germany). PCR was performed on an ABI Q5 real-time PCR system. The primers used in this study are shown in Table S2.

### 2.10. Western Blotting

Cells and liver samples were lysed with lysis buffer containing protease and phosphatase inhibitor (Solarbio), and lysates were loaded onto SDS-PAGE and transferred to a PVDF membrane (Millipore, Beijing, China). The blotted membrane was probed with antibodies against antibodies against MBOAT2 (121453; Abcam, Cambridge, MA, USA), proliferator-activated receptor α (PPARα, AF5301; Affinity, Inc., Fairborn, OH, USA), PPARγ (GB11164; Servicebio, Inc. Wuhan, China), sterol regulatory-element binding protein 1 (SREBP1, GB11524; Servicebio, Inc. Wuhan, China), and β-actin (8227; Abcam, Cambridge, MA, USA), and visualized using the chemiluminescence imaging system (CLINX, 6100, Clinx Science Instruments Co. Ltd., Shanghai, China).

### 2.11. Untargeted Metabolomic Analysis

Untargeted metabolomic analysis of supernatants of *L. salivarius* SNK-6 culture media was performed using a liquid chromatography–mass spectrometry (LC-MS/MS) system with a high-resolution mass spectrometer Q Exactive HF (Thermo Fisher Scientific, Sunnyvale, CA, USA). The chromatographic separation of all samples was performed on an ACQUITY UPLC BEH C18 (1.7 μm, 2.1 × 100 mm, Waters, Milford, LA, USA), as described previously [22]. Compound Discoverer 3.1 (Thermo Fisher Scientific, Waltham, MA, USA) software was used for LC-MS/MS data processing, as described previously [23]. Taxonomic and functional annotations were performed on the identified metabolites against the Human Metabolome Database. The functional annotation of pathways was performed using the KEGG PATHWAY database. Raw data are deposited in MetaboLights (www.ebi.ac.uk/metabolights/MTBLS5392 (accessed on 15 July 2022)) with assess number: MTBLS5392.

### 2.12. SCFA Quantification

Short-chain fatty acids (SCFA) quantification was performed on an ABSCIEX 6500+ QTRAP LC-MS/MS System (SCIEX, Foster City, CA, USA) and chromatographic separation was accomplished on an ACQUITY Premier BEH C8 Column, 1.7 µm, 2.1 mm × 50 mm column (Waters) at 40 °C. MultiQuant software (SCIEX, Framingham, MA, USA) was used for data analysis.

### 2.13. Bile Acid Composition

Bile acids (BAs) were assayed on an LC–MS/MS system consisting of an ACQUITY UPLC I-Class System (Waters) coupled with a QTRAP 6500 LC-MS/MS System (SCIEX). Chromatographic separation was performed on a Waters ACQUITY BEH C18 Column, 1.7 µm, 2.1 mm × 50 mm column. Automatic identification and integration of each MRM transition with default parameters in Analyst software (SCIEX) was used for BA assays, as described previously [24].

### 2.14. Amino Acid Composition

Amino acids (AAs) were assayed using the Sciex5500- Transcend II (Thermo Fisher Scientific) LC–MS/MS system. Chromatographic separation was accomplished using a 100 mm×2.1 mm HSS T3 column (Waters). Chromatographic peak extraction and quantitative analysis were performed using MultiQuant software (SCIEX, v3.0.3 HotFix 3).

### 2.15. Statistical Analysis

SPSS 16.0 was used for statistical analysis using a One-Way ANOVA and Tukey’s HSD comparisons. For two group analysis, a dependent sample *t*-test was used when the data of two groups conformed to a normal distribution, otherwise the nonparametric Mann–Whitney test was used. GraphPad Prism 6 (La Jolla, CA, USA) was used to draw the figures. The data are presented as the mean ± SEM. A *p* < 0.05 was considered significant.

## 3. Results

### 3.1. Genome Sequencing of L. salivarius SNK-6

As shown in Figure S1 and Table 1, the genome sequence of *L. salivarius* SNK-6 comprised a circular chromosome of 1,723,288 bp and plasmid 1 (204,234 bp) and plasmid 2 (26,769 bp), resulting in GC content of 33.03%, similar to that of plasmid 2 (32.79%), but higher than that of the plasmid 1 (31.93%). The chromosome contained 1597 putative coding sequences, 78 tRNA genes and 22 rRNA genes (Table 1). Tetracycline resistance genes, frequently encountered in *Lactobacillus*, were also found in *L. salivarius* SNK-6 (Table S3). Phylogenetic analysis indicated that the *Lactobacillus* isolate used in this study belonged to a subclade of *L. salivarius*, showing 99.5% similarity to *Lactobacillus salivarius* BCRC 14659 (Figure S2).

### 3.2. L. salivarius SNK-6 Alleviates Fat Deposition in the NAFLD Model in Laying Hens

**Figure 1:**
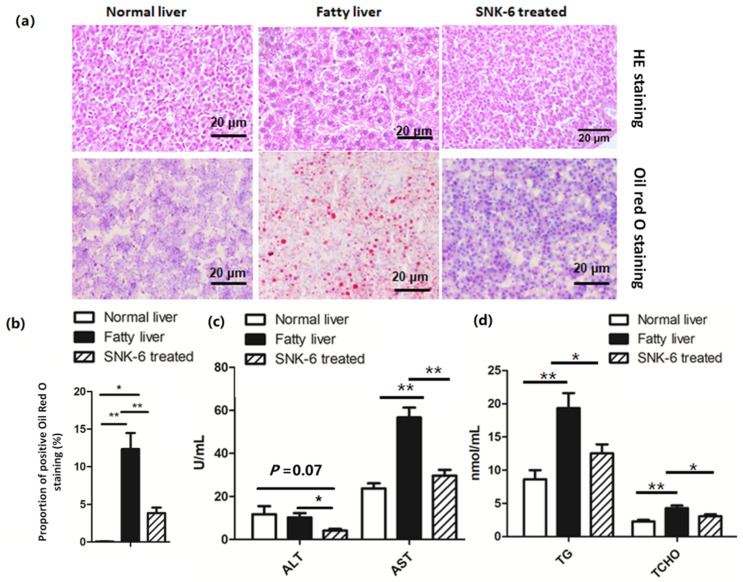
*L. salivarius* SNK-6 attenuates liver fat deposition: (**a**) HE and Oil Red staining (200 × magnification); (**b**) Percentage of Oil Red O staining positive area in liver; (**c**) Serum transaminase activity analysis; (**d**) Serum TG and TCHO content analysis (n = 15/group). Note: TG, triglyceride; TCHO: total cholesterol; ALT, alanine aminotransferase; AST, aspartate aminotransferase. * *p* < 0.05 and ** *p* < 0.01.

As shown in Figure 1, we found that serum TG and TCHO levels and the activity of AST were significantly increased (*p* < 0.01, Figure 1c,d) in the fatty liver group, when compared to the control normal liver group. *L. salivarius* SNK-6 significantly reduced liver fat deposition (Figure 1a,b), serum TG and TCHO levels (Figure 1d, *p* < 0.05), and the activity of AST and ALT (Figure 1c, *p* < 0.05) in laying hens when compared to the fatty liver group. In addition, the area of fat droplet was significantly decreased (*p* < 0.01, Figure 1b) in *L. salivarius* SNK-6 treated laying hens (Figure 1c), when compared to the fatty liver group. However, the area of Oil red O positive staining in the liver of *L. salivarius* SNK-6 treated group was still higher than that in the control normal liver group (Figure 1b, *p* < 0.05).

### 3.3. miR-130a-5p Targets the MBOAT2/ PPAR/SREBP Pathway

**Figure 2:**
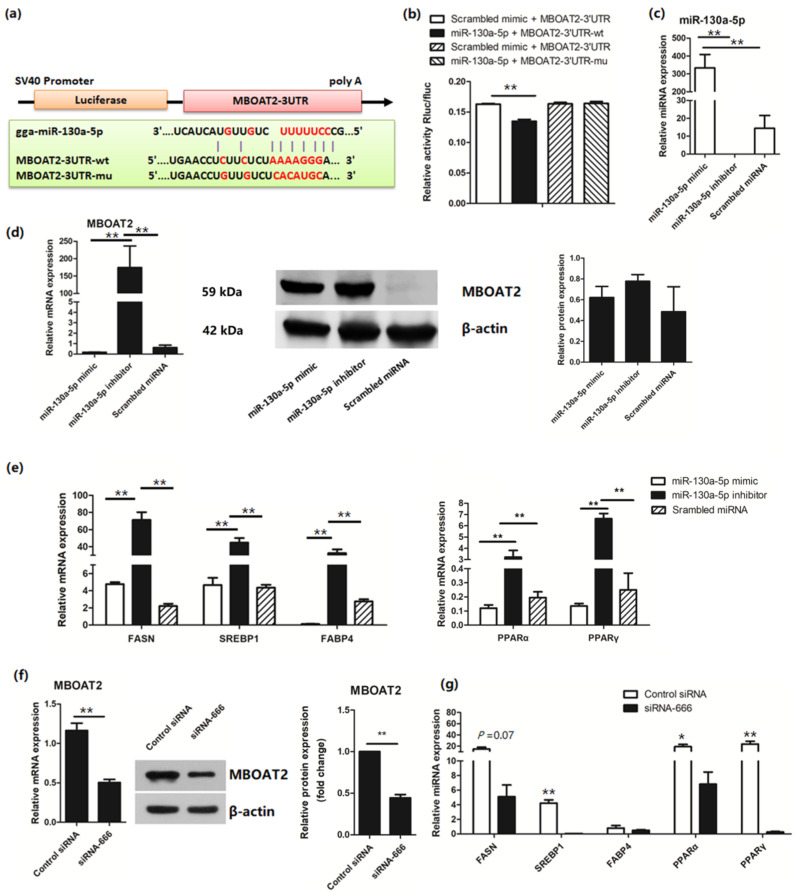
miR-130a-5p is a lipid metabolism related miRNA: (**a**) Wild-type and mutant sequences of MBOAT2; (**b**) Dual luciferase reporter gene assay; (**c**) Expression of miR-130a-5p in cells after miR-130a-5p mimic or inhibitor transfection; (**d**) mRNA and protein levels of MBOAT2 after up-regulation or down-regulation of the miR-130a-5p; (**e**) Relative mRNA levels of genes involved in the PPAR/SREBP pathway in LMH cells transfected with miR-130a-5p mimics or inhibitor; (**f**) Validation of the siRNA for silencing of MBOAT2 by RT-qPCR and Western blotting analysis; (**g**) RT-qPCR analysis of genes associated with the PPAR/SREBP pathway in MBOAT2-inhibited cells. The results are presented as the mean ±SEM of at least triplicate experiments. * *p* < 0.05 and ** *p* < 0.01.

We previously found that miR-130a-5p was significantly downregulated in the liver laying hens with fatty liver, compared to the control group by miRNA sequencing [18]. Potential targets of miR-130a-5p were predicted by targetScan and miRnada and showed that the TG-synthesis-related gene *MBOAT2* may be targeted by miR-130a-5p (Table S4), suggesting that miR-130a-5p might be a lipid metabolism related miRNA. Then, the binding sites for *MBOAT2* mRNAs of the miR-130a-5p were cloned into a luciferase reporter vector (Figure 2a). We confirmed that transfection with miR-130a-5p mimic reduced the luciferase activity compared to the scrambled miRNA mimics (Figure 2b). Overexpression of miR-130a-5p suppressed mRNA of MBOAT2, and downregulation of miR-130a-5p reversed the results of MBOAT2 (Figure 2c,d), suggesting that *MBOAT2* is a direct target of miR-130a-5p. However, no significant decrease in MBOAT2 protein expression was observed in miR-130a-5p enhanced cells.

Subsequently, mRNA expression of genes associated with the PPAR/SREBP pathway was investigated to further determine the regulatory role of miR-130a-5p in lipid metabolism. Inhibition of miR-130a-5p significantly increased the mRNA levels of *PPARα*, *PPARγ*, fatty acid binding protein 4 (*FABP*4), *SREBP1,* and fatty acid synthase (*FASN*), proving that miR-130a-5p was involved in the regulation of lipid metabolism (Figure 2e). However, we did not find a significant decrease in the mRNA levels of *PPARα*, *PPARγ*, *SREBP1*, *FABP4*, and *FASN* in the cells after miR-130a-5p upregulation. To confirm the relationship between MBOAT2 and the PPAR/SREBP pathway, siRNAs were designed and optimized to silence *MBOAT2* (Figure 2f). Treatment of LMH cells with the MBOAT2 siRNA led to downregulation of *SREBP1*, *FASN*, *PPARα*, and *PPARγ* (Figure 2g), which was in contrast to the results of miR-130-5p downregulation in LMH cells.

### 3.4. L. salivarius SNK-6 Regulates the miR-130a-5p/MBOAT2 Pathway in the NAFLD Model

**Figure 3:**
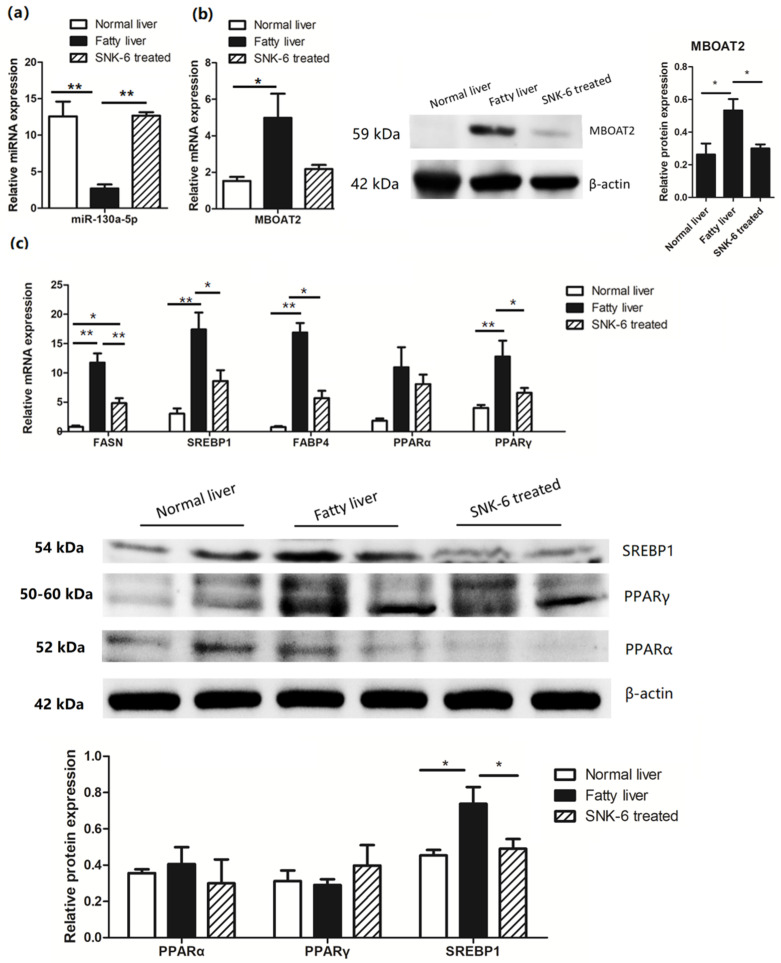
*L. salivarius* SNK-6 regulates the miR-130a-5p/MBOAT2 pathway: (**a**) Expression of miR-130a-5p; (**b**) The mRNA and protein levels of MBOAT2 in the liver of *L. salivarius* SNK-6 treated layers; (**c**) RT-qPCR and Western blotting analysis of genes associated with the PPAR/SREBP pathway. The results are presented as the mean ±SEM of at least triplicate replicates. * *p <* 0.05 and ** *p <* 0.01.

We further determined the role of *L. salivarius* SNK-6 in lipid metabolism in the NAFLD model in laying hens. Expression of miR-130a-5p, MBOAT2, and PPAR/SREBP pathway-related genes was investigated by RT-qPCR. Comparing to the fatty liver group, *L. salivarius* SNK-6 significantly (*p* < 0.01) upregulated the expression of hepatic miR-130a-5p (Figure 3a) but suppressed mRNA and protein expression of MBOAT2 (Figure 3b), although no significant difference was observed in the mRNA expression. Supplementation with *L. salivarius* SNK-6 inhibited the mRNA expression of fat-synthesis-related genes *SREBP1*, *FASN*, and *FABP4*, as well as the lipolytic gene *PPARγ* (Figure 3c, *p <* 0.05). This was consistent with the result observed in LMH cells with *MBOAT2* inhibition (Figure 2e,g). In addition, the protein expression of SREBP1 was obviously down-regulated in the liver of *L. salivarius* SNK-6 treated layers, compared to the fatty liver group (Figure 3c, *p* < 0.05).

### 3.5. L. salivarius SNK-6 Culture Medium Inhibits fat Deposition in Hepatocytes

**Figure 4:**
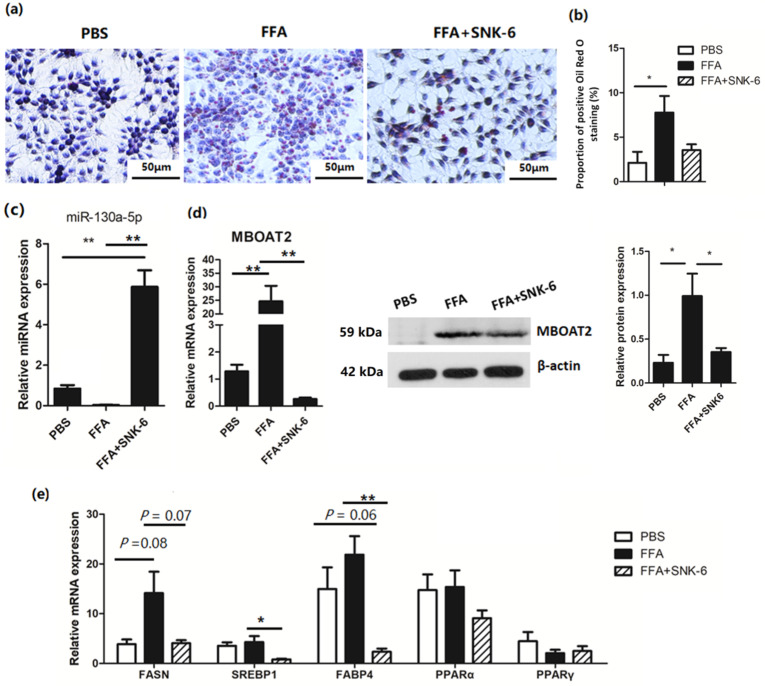
*L. salivarius* SNK-6 metabolite inhibits FFA-induced lipid deposition in hepatocytes: (**a**) Oil red O staining (200× magnification).; (**b**) Percentage of Oil Red O staining positive area in cells; (**c**) Expression of miR-130a-5p in *L. salivarius* SNK-6 metabolite treated hepatocytes; (**d**) The mRNA and protein levels of MBOAT2 in *L. salivarius* SNK-6 metabolite treated hepatocytes; (**e**) qRT-PCR analysis of genes associated with the PPAR/SREBP pathway. The results are presented as the mean ±SEM of at least triplicate experiments. * *p* < 0.05 and ** *p* < 0.01.

To investigate how *L. salivarius* SNK-6 regulated lipid metabolism, an FFA-induced hepatocyte steatosis model was established, and the supernatant of *L. salivarius* SNK-6 culture medium was added to the cell model. After co-cultivation for 24 h, Oil Red O staining showed that the culture medium of *L. salivarius* SNK-6 inhibited fat deposition in the cells (Figure 4a) with a decrease in trend in the area of fat droplet, compared to the FFA treated cells (Figure 4b), although no statistical difference was observed. *L. salivarius* SNK-6 treatment upregulated the expression of miR-130a-5p (Figure 4c), but downregulated mRNA and protein expression of MBOAT2 (Figure 4d), comparing to the FFA treated LMH cells. We also found that FFA activated the PPAR/SREBP pathway by upregulating the expression of fat-synthesis-related gene *FASN*, while *L. salivarius* SNK-6 culture medium suppressed the mRNA expression of *FASN*, *SREBP1*, and *FABP4* (Figure 4e).

### 3.6. Metabolite Analysis of L. salivarius SNK-6

**Figure 5:**
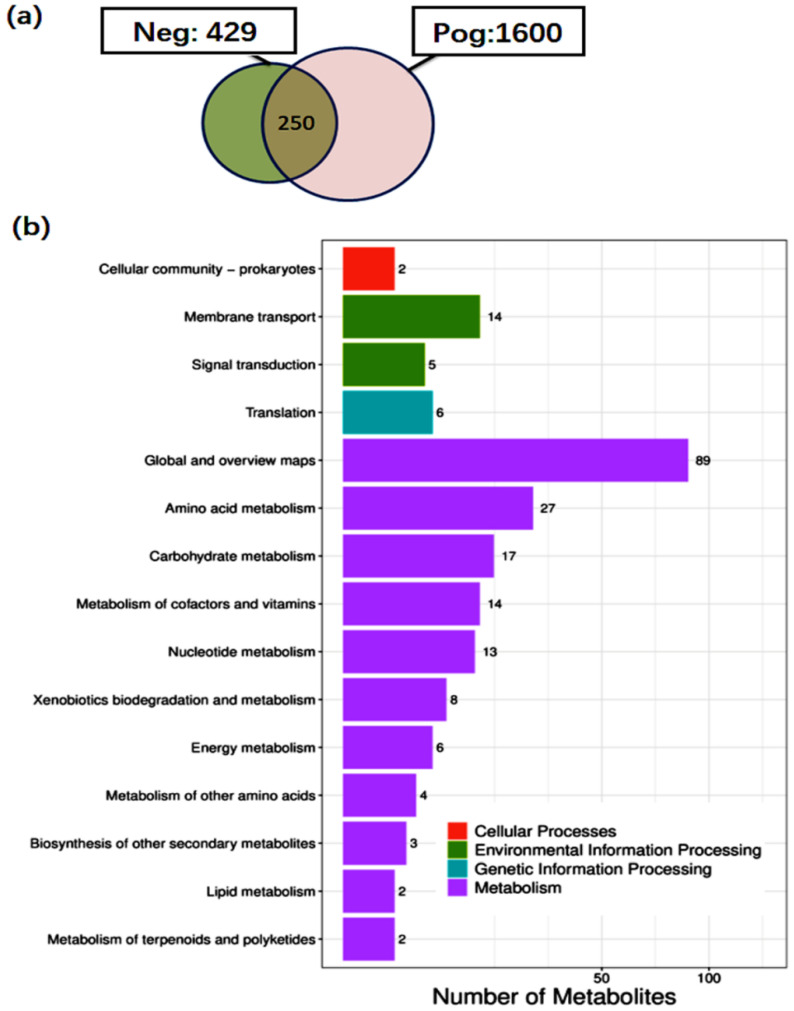
Untargeted metabolome analysis of *L. salivarius* SNK-6 culture media: (**a**) Number of metabolites quantified by LC-MS/MS; (**b**) KEGG enrichment analysis of the metabolites.

We further analyzed the metabolites of *L. salivarius* SNK-6 by untargeted metabolome analysis using UHPLC-MS/MS, and 250 metabolites were quantified in the supernatants of *L. salivarius* SNK-6 culture medium (Table S5, Figure 5a), including two amino acids, 24 fatty acyls, six indoles and derivatives, 53 carboxylic acids and derivatives, eight benzene and substituted derivatives, 13 organooxygen compounds, and four pyrimidine nucleosides. To determine the main metabolic pathways correlated with the metabolites of *L. salivarius* SNK-6, KEGG enrichment analysis was performed. Figure 5b shows that these metabolites were scattered into multiple metabolic pathways, including global and overview maps, AA metabolism, carbohydrate metabolism, metabolism of cofactors, nucleotide metabolism, and lipid metabolism. Glutaric acid is involved in FA degradation, β-muricholic acid participates in secondary BA biosynthesis, and 10 metabolites including pyruvic acid, succinate, fumaric acid, trans-cinnamic acid, salicylic acid, (e)-*p*-coumaric acid, 4-toluic acid, phthalic acid, acetophenone, and 3-coumaric acid were associated with degradation of aromatic compounds (Table S6).

**Figure 6:**
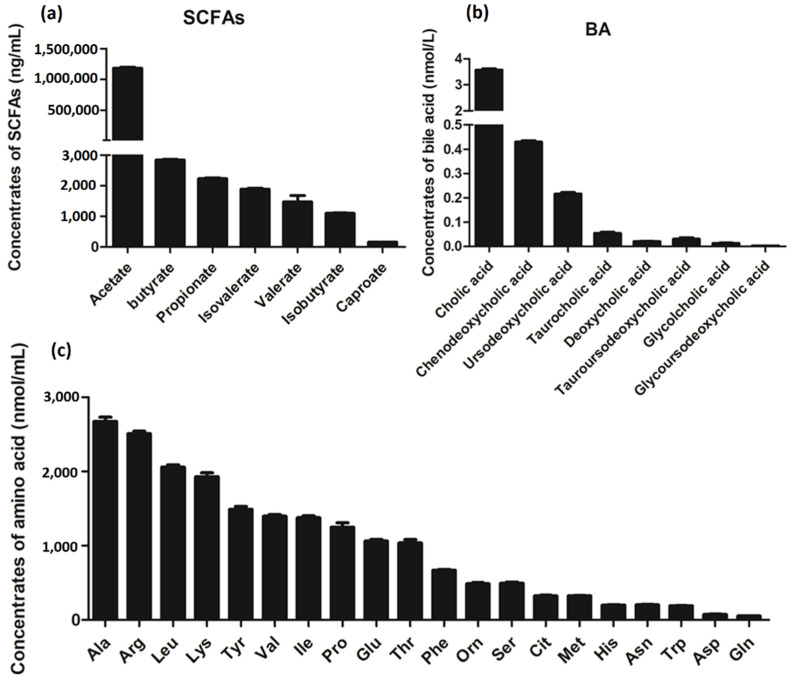
Targeted metabolome analysis of supernatants of *L. salivarius* SNK-6 culture media. (**a**) SCFAs. (**b**) Bile acid. (**c**) Amino acid, n = 6.

Subsequently, targeted metabolome analysis was performed to qualify the SCFAs, BAs, and AAs in the supernatants of *L. salivarius* SNK-6 culture media. As shown in Figure 6, the main components of SCFAs including acetate, butyrate, and propionate, while Cholic acid (CA), Ursodeoxycholic acid (UDCA), Chenodeoxycholic acid (CDCA), and tauroursodeoxycholic acid (TCA) are the four most enriched BAs of *L. salivarius* SNK-6 metabolites. The most enriched AAs including Ala, Arg, Leu, Try, and Val were measured in the supernatants of *L. salivarius* SNK-6 culture media.

## 4. Discussion

In this study, we screened a new probiotic strain, *L. salivarius* SNK-6, which was isolated from the ileum of Xinyang black-feather laying hens and had properties of inhibiting fat deposition in vitro and in vivo. We found that oral *L. salivarius* SNK-6 treatment could reduce liver fat deposition, serum TG and TCHO levels, as well as the activity of AST and ALT in the NAFLD model of laying hens. Furthermore, it also had an effect on the regulation of the genes involved in the miR-130a-5p/MBOAT2 pathway, including *MBOAT2*, *FASN*, *SREBP1*, *FABP4*, and *PPARγ*, suggesting that oral *L. salivarius* SNK-6 treatment could alleviate NAFLD.

The role of miRNAs in liver lipid metabolism and inflammatory response has been confirmed, and regulating the expression of these miRNAs can help reduce the occurrence and development of fatty liver [25]. Studies have shown that miR-130a-5p is significantly decreased in liver cancer tissues, and up-regulation of miR-130a inhibits the proliferation, migration, and invasion of liver cancer cells [26]. MBOAT2 is closely related to the formation of TG [27]. We found that miR-130a-5p was downregulated in the liver of laying hens with fatty liver, targeting the expression of MBOAT2. *L. salivarius* SNK-6 treatment upregulated liver miR-130a-5p, inhibited expression of MBOAT2, and reduce liver fat deposition, indicating that *L. salivarius* SNK-6 can regulate the liver fat metabolism partly by targeting miR-130a-5p/MBOAT2 signaling axis. SREBP1 is a lipid lipogenic gene that regulates adipogenesis by activating genes involved in FA and TG synthesis [28]. Peroxisome proliferator-activated receptors (PPARs) are key metabolic regulators in the liver and are important targets for NAFLD therapy [29]. Fat-specific deletion of PPARγ results in severe atrophy of fat, highlighting the role of PPARγ in adipocyte development [30]. PPARγ/SREBP-1c pathways are involved in alleviation of Antrodan-treated NAFLD via upregulated pAMPK and Sirt1, and downregulated PPARγ and SREBP-1c, thereby suppressing lipid biosynthesis and facilitating insulin resistance [31]. In this study, we found that inhibition of miR-130a-5p or MBOAT2 regulated expression of genes related to the MBOAT2/PPAR/SREBP signaling pathway. Furthermore, oral *L. salivarius* SNK-6 treatment upregulated miR-130a-5p, inhibited expression of MBOAT2, FASN, PPARγ, and SREBP1, which suggested that *L. salivarius* SNK-6 regulated liver lipid metabolism partly through miR-130a-5p/MBOAT2 signaling. Based on these findings, the inhibition of MBOAT2 activity or up-regulation of miR-130a-5p is of utmost importance when designing liver-protective therapies to attenuate fatty liver disease. However, we did not find a significant decrease in the mRNA levels of *PPARα*, *PPAR*γ, *SREBP1*, *FABP4*, and *FASN* in the cells after miR-130a-5p overexpression, which may due to the limitations of miRNAs, including one miRNA can target numerous genes, off-target effects, and inefficient of intracellular delivery. Whether miR-130a-5p could modulate lipid metabolism via other receptors in a manner dependent on the PPAR/SREBP pathway is far from clear. Notably, in addition to the protein size recommended by the manufacturer, we also found two bands appropriately 75 kDa and 45 kDa, the expression trend of which were similar to the predicted 59 kDa of MBOAT2. Whether this is associated with non-specific binding or post-translational modification needs further determination, since the polyclonal antibody of MBOAT2 (Abcam, 121453) was the only antibody currently available.

SCFAs are bacterial fermentation products of indigestible foods, which are crucial for intestinal health. Oral supplementation of SCFAs is beneficial for NAFLD treatment by reducing steatosis and inflammation [32,33]. Acetate, butyrate, and propionate are the main SCFAs, and are important signaling molecules regulating lipid metabolism genes, including activating hepatic AMPK phosphorylation and altered expression of its downstream target genes such as *PPARa*, *SREBP1*, and *FFAR2* [32,34,35]. Additionally, some bacteria that produce butyrate, acetate, and propionate have been shown to modulate hepatic lipid metabolism; for example, acetate-producing *Desulfovibrio vulgaris* has been shown to attenuate hepatic steatosis [36]. Butyrate-producing *Lactobacillus* and *Bifidobacterium* alleviate lipid accumulation and inflammation in NAFLD rats [37]. In the present study, we identified 250 metabolites in the supernatants of *L. salivarius* SNK-6 culture media. Most of them participated in multiple metabolism-related pathways, including AA and lipid metabolism. We also demonstrated that butyrate, acetate, and propionate were the main SCFAs in the culture media of *L. salivarius* SNK-6. Acetate was the most common component, suggesting that *L. salivarius* SNK-6-derived acetate mediates the crosstalk between *L. salivarius* SNK-6 and liver metabolism.

BAs have important roles in regulating lipid, glucose and energy metabolism, and are crucial to protect liver cells from cholesterol and BA toxicity [38]. BAs contain a variety of CA derivatives, each of which exhibits a specific affinity for BA receptors and acts as a signaling molecule with unique effects to modulate hepatic lipid metabolism [39]. ER stress is enhanced in obesity [40], and it is involved in NAFLD. CA is reported to reduce ER stress in cultured hepatoma cells and adipocytes [41] and acts as an antibacterial agent in the gut that controls fungal overgrowth by degradation of fungal biofilms [42]. UDCA is used for the treatment of primary biliary cirrhosis, which activates PKC and MAPK signaling and anti-inflammatory hepatocyte pathways, as well as promoting bile HCO_3_^−^ secretion to reduce cholestasis and reduce liver damage [39,43,44]. CDCA has been obtained in mice on a high-fat diet, and shown to reduce weight and improve glucose tolerance [45]. TCA has recently been reported to attenuate hepatic steatosis, gut inflammatory responses, and insulin resistance in mice [46]. Here, we found that CA, UDCA, CDCA, and TCA were the four most-enriched BAs among *L. salivarius* SNK-6 metabolites. Combined with the beneficial effects of *L. salivarius* SNK-6 treatment on hepatic fat deposition that we observed in this study, it can be speculated that the beneficial effects of *L. salivarius* SNK-6 on the liver may be regulated by the metabolites of *L. salivarius* SNK-6. Further analysis indicated that *L. salivarius* SNK-6 treatment significantly suppressed the expression of hepatic FASN, FABP4, and SREBP1. All these data collectively indicated that *L. salivarius* SNK-6 treatment prevented NAFLD partly through suppressing de novo FA synthesis in the liver. However, the specific mechanisms of *L. salivarius* SNK-6, including pathways and the major metabolites that participate in the regulatory of lipid metabolism, still need to be further confirmed.

## 5. Conclusions

This study confirmed that *L. salivarius* SNK-6 had a potential reparative effect in a chicken model of NAFLD. MBOAT2 is a direct target of miR-130a-5p. *L. salivarius* SNK-6 alleviated fat deposition in the liver by upregulating miR-130a-5p and inhibiting expression of MBOAT2. Downregulation of MBOAT2 or *L. salivarius* SNK-6 supplementation in vivo and in vitro suppressed the PPAR/SREBP pathway, suggesting that *L. salivarius* SNK-6 alleviated liver damage partly via the miR-130a-5p/MBOAT2 signaling pathway. In addition, acetate, butyrate, and propionate are the most abundant SCFAs, while CA, UDCA, CDCA, and TCA are the four most enriched BAs among *L. salivarius* SNK-6 metabolites, which may have contributed to the reparative effect of *L. salivarius* SNK-6 in the chicken model of NAFLD. These results suggest a new therapeutic target for NAFLD.

## Figures and Tables

**Table 1:** Summary of the raw data.

Item	Chr 1	Plasmid 1	Plasmid 2
Genome Length	1,723,288 bp	204,234 bp	26,769 bp
Number of scaffold(contig)	1	1	1
GC content	33.00%	31.90%	32.79%
Num of CDS	1587	199	29
tRNA	78		
rRNA	22		

## Data Availability

All data supporting our findings are included in the manuscript.

## Supplementary Materials

The following supporting information can be downloaded at https://www.mdpi.com/article/10.3390/cells11244133/s1, Figure S1: The genome sequence of *L. salivarius* SNK-6; Figure S2: Phylogenetic analysis *L. salivarius* SNK-6; Table S1: List of miRNA mimics/inhibitor/siRNA used in the study; Table S2: List of the primers used for RT-qPCR analysis in the study; Table S3: Antibiotic resistance analysis; Table S4: Potential targets of gga-miR-130a-5p; Table S5: list of identified metabolites; Table S6: KEGG analysis of metabolites.
