# Supplementary material for: Lactobacillus salivarius SNK-6 Regulates Liver Lipid Metabolism Partly via the miR-130a-5p/MBOAT2 Pathway in a NAFLD Model of Laying Hens
Source: Cells. 2022 Dec 19;11(24):4133. doi: 10.3390/cells11244133 (PMC9776975; doi:10.3390/cells11244133)

Supplementary Figures:

Figure S1 The genome sequence of *L. salivariu* SNK-6

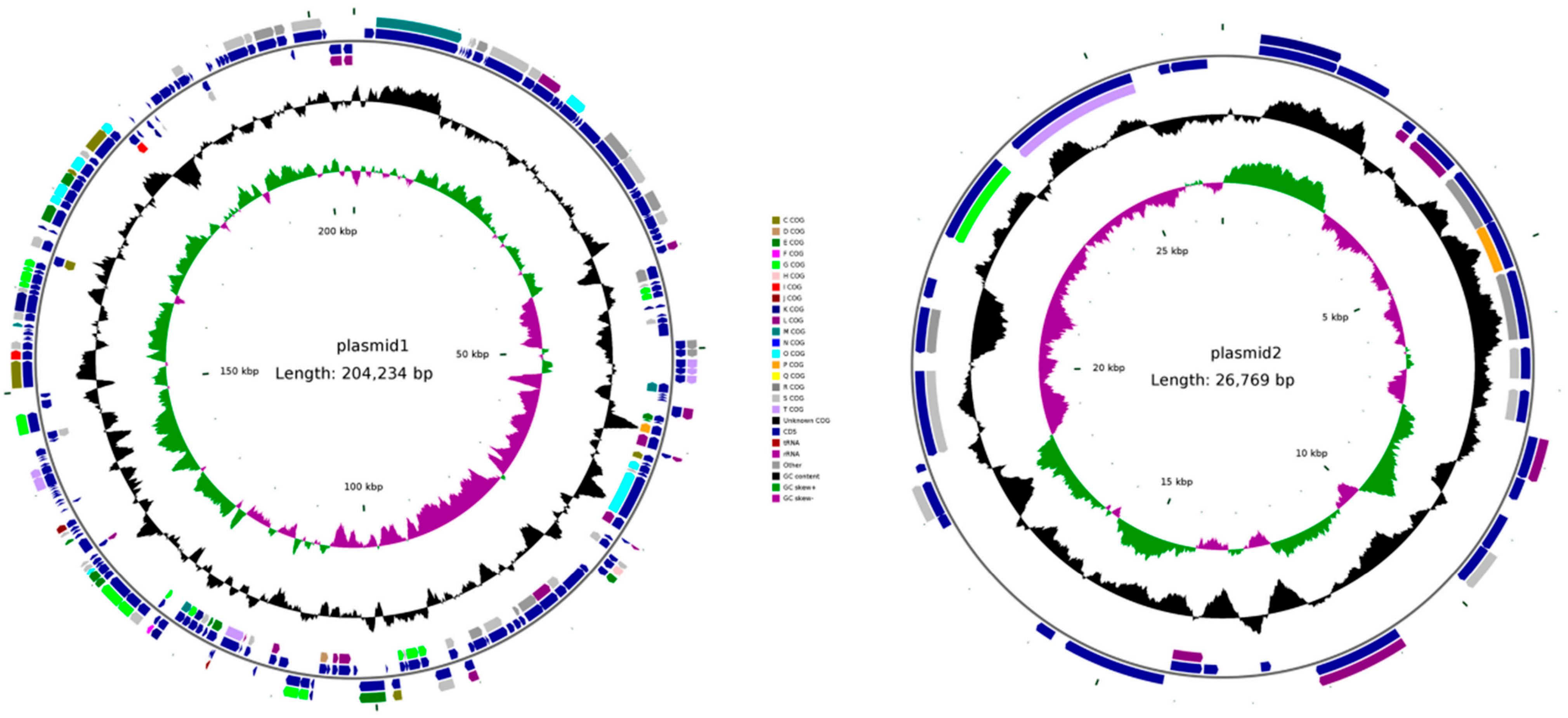

Figure S2. Phylogenetic analysis *L. salivariu* SNK-6

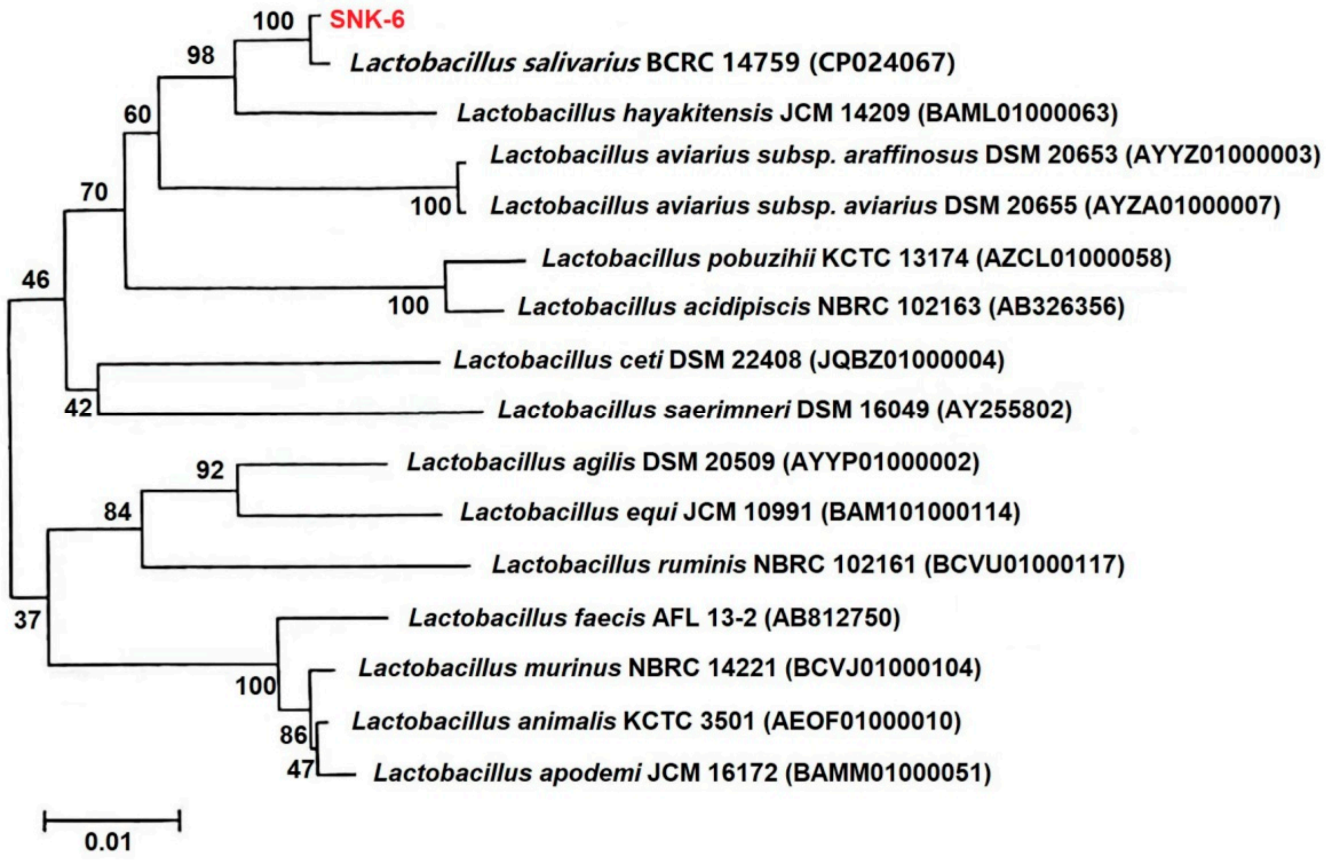

Supplement: Supplementary file 1 [file cells-11-04133-s001.zip › Supplementary Figures.pdf]
